# Bonus versus penalty: How robust are the effects of contract framing?

**DOI:** 10.1007/s40881-017-0039-9

**Published:** 2017-09-23

**Authors:** Jonathan de Quidt, Francesco Fallucchi, Felix Kölle, Daniele Nosenzo, Simone Quercia

**Affiliations:** 1 Institute for International Economic Studies, Stockholm University, Stockholm, Sweden; 2 CESifo, Munich, Germany; 3 Luxembourg Institute of Socio-Economic Research, Esch-sur-Alzette, Luxembourg; 4 Center for Social and Economic Behavior, University of Cologne, Cologne, Germany; 5 School of Economics, University of Nottingham, Nottingham, UK; 6 Institute for Applied Microeconomics, University of Bonn, Bonn, Germany

**Keywords:** Contract framing, Bonus, Penalty, Fine, Loss aversion, C9, D03, J24

## Abstract

We study the relative effectiveness of contracts that are framed either in terms of bonuses or penalties. In one set of treatments, subjects know at the time of effort provision whether they have achieved the bonus/avoided the penalty. In another set of treatments, subjects only learn the success of their performance at the end of the task. We fail to observe a contract framing effect in either condition: effort provision is statistically indistinguishable under bonus and penalty contracts.

**Electronic supplementary material:**

The online version of this article (doi:10.1007/s40881-017-0039-9) contains supplementary material, which is available to authorized users.

## Introduction

Although incentive pay can be very effective in raising employees’ performance (e.g., Lazear 2000), the way incentives are described also matters. A recent experimental literature suggests that incentives are more effective when they are framed as penalties for poor performance rather than bonuses for good performance. For example, Hannan et al. (2005) found that employees exerted significantly more effort under a “penalty” contract that paid a base salary of $30 minus a $10 penalty if they did not meet a performance target, than under a “bonus” contract that paid $20 plus a bonus of $10 if the target was met. The two contracts are isomorphic and so the increase in effort is entirely due to a *framing effect* (Tversky and Kahneman 1981). Several other studies confirmed this finding, both in the lab (Armantier and Boly 2015; Imas et al. 2017) and in the field (Fryer et al. 2012; Hossain and List 2012; Hong et al. 2015).

**Fig. 1:**
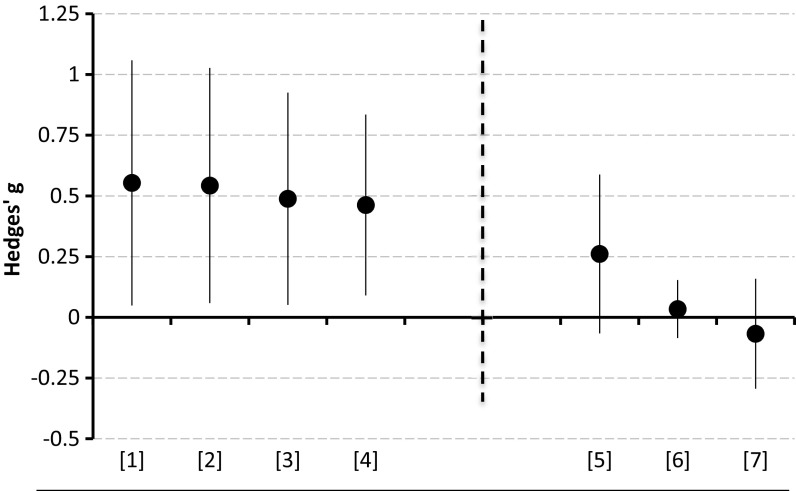
Effect size of contract framing in previous experiments. Effect sizes are computed using Hedges’ *g* (Hedges 1981). Bars represent 95% confidence intervals computed as $g \pm 1.96*\left\{ {\sqrt {\left[ {\frac{{n_{p} + n_{b} }}{{n_{p} n_{b} }} + \frac{{g^{2} }}{{2(n_{p} + n_{b} )}}} \right]} } \right\}$.* [1]*  Armantier and Boly (2015)—Burkina Faso; *[2]* Hannan et al. (2005); *[3]* Imas et al. (2017); *[4]* Armantier and Boly (2015)—Canada; *[5] * Brooks et al. (2012); *[6]* DellaVigna and Pope (2016); *[7]*  Grolleau et al. (2016)

The size of this framing effect is large. Figure 1 (left panel) shows effect sizes and confidence intervals of the three lab experiments cited above (Hannan et al. 2005; Armantier and Boly 2015; Imas et al. 2017).^1^ The average Hedges’ *g* statistic across these studies is 0.51 (Hedges 1981).^2^ However, Fig. 1 (right panel) also shows that three further studies found considerably smaller effects, which are either statistically insignificant (DellaVigna and Pope 2016; Grolleau et al. 2016), or only marginally significant (Brooks et al. 2012).

One systematic difference between the experiments in the left and right panels of Fig. 1 relates to whether or not subjects could check during the task whether they had met the performance target and hence their monetary compensation. In Brooks et al. (2012), DellaVigna and Pope (2016), Grolleau et al. (2016), subjects were told in advance what the target was and could verify their monetary compensation at any point during the experiment.^3^ This is not the case for the other studies in Fig. 1.^4^ However, there are many other differences across these studies, which makes it difficult to draw definitive conclusions about the exact causes of the discrepancy in effect sizes (Table A1 in electronic supplementary material summarizes the characteristics of the studies included in Fig. 1). In this paper, we report an experiment designed to replicate the pattern displayed in Fig. 1 by testing whether the effectiveness of contract framing depends on the availability of information about the performance target.

We describe our experiment design in Sect. 2. Subjects performed a real-effort task under either a bonus or penalty contract. Both contracts specified a base pay and an extra amount of money that subjects could earn by meeting a performance target. In the bonus contract, subjects were told that they could increase their base pay by meeting the target. In the penalty contract, they were told that the base pay would be reduced if they missed the target. We implemented these contracts under two conditions. In one condition, akin to the studies in the left panel of Fig. 1, the performance target was not specified ex-ante: subjects were told that their performance would be compared with the average performance in a previous experiment. In the other condition we announced the target at the beginning of the task, as in the studies in the right panel of Fig. 1.

We report our results in Sect. 3. Performance in the real-effort task is statistically indistinguishable under the bonus and penalty contracts, both under announced and unannounced targets. While the absence of contract framing effects under announced targets is consistent with the existing evidence, our experiment fails to replicate the findings of the studies displayed on the left panel of Fig. 1 that had found significant framing effects when the target was unannounced. We discuss the implications of these results in Sect. 4.

## Experimental design

Our experiment was conducted online with 853 subjects recruited on Amazon’s Mechanical Turk (MTurk).^5^ The experiment consisted of 3 parts plus a questionnaire. Subjects knew this in advance, although they did not receive instructions for each part until they had completed the previous ones. Only one part, randomly selected at the end, was paid out.

In Part 1, subjects participated in the “Encryption Task” (Erkal et al. 2011): they had to encode a series of words by substituting letters with numbers using predetermined letter-to-number assignments. Subjects had 5 min to encode as many words as possible and were paid $0.05 per word, while receiving live feedback about the total number of words encoded so far.^6^ This part of the experiment was the same across treatments and is used to obtain a baseline measurement of subjects’ ability in the task.

Part 2 varied across treatments according to a 2 × 2 between-subject design. In all treatments, subjects had again to encode words and were paid based on how many words they encoded within 10 min, again with live feedback on the total number of words encoded. In the *Bonus* treatments the payment specified a base pay of $0.50 plus a bonus of $1.50 if the subject encoded as many words as specified in a productivity target. In the *Penalty* treatments the payment specified a base pay of $2.00 minus a penalty of $1.50 if the target was not met. In the *Announced* treatments the target was announced at the beginning of the task.^7^ In the *Unannounced* treatments the target was left unspecified: subjects were just told that the target was based on the average productivity of participants in a previous study.^8^

Part 3 was again the same in all treatments. One of the explanation for the existence of contract framing effects suggested in the literature is loss aversion. To assess the role of loss aversion in our experiment, we used the lottery choice task introduced by Gächter et al. (2010). Subjects received a list of six lotteries and decided, for each lottery, whether to accept it (and receive its realization as a payment) or reject it (and receive nothing). Each lottery specified a 50% probability of winning $1.00 and a 50% probability of losing an amount of money that varied across lotteries from $0.20 to $1.20, in $0.20 increments.^9^ As discussed by Gächter et al. (2010), a subject’s pattern of acceptances/rejections in this task measures his/her degree of loss aversion.

Finally, subjects completed a short questionnaire measuring standard socio-demographics. Additionally in the Unannounced treatments subjects were asked to guess what the target was before learning the outcome of the experiment.^10^

Table 1 summarizes the design.^11^ Sample sizes were determined using power analysis. Based on the average effect size (*g* = 0.51) reported across the three studies with unannounced targets, we assigned 137 observations to each of the Bonus and Penalty treatments in the Unannounced condition. This gives us 98% power to detect the original average effect size at the 5% level of significance. We assigned our remaining resources to recruit subjects in the Announced condition. Given the resulting sample size (292 subjects in Bonus; 287 in Penalty), we have an 80% power to detect an effect size of at least 0.24 at a 5% level of significance.^12^

**Table 1:** Experiment design

	Unannounced treatment	Announced treatment
Bonus treatment	Contract pays $0.50 + $1.50 if subject meets an unspecified performance target (*N* = 137)	Contract pays $0.50 + $1.50 if subject meets a pre-specified performance target (*N* = 292)
Penalty treatment	Contract pays $2.00 − $1.50 if subject does not meet an unspecified performance target (*N* = 137)	Contract pays $2.00 − $1.50 if subject does not meet a pre-specified performance target (*N* = 287)

## Results

**Fig. 2:**
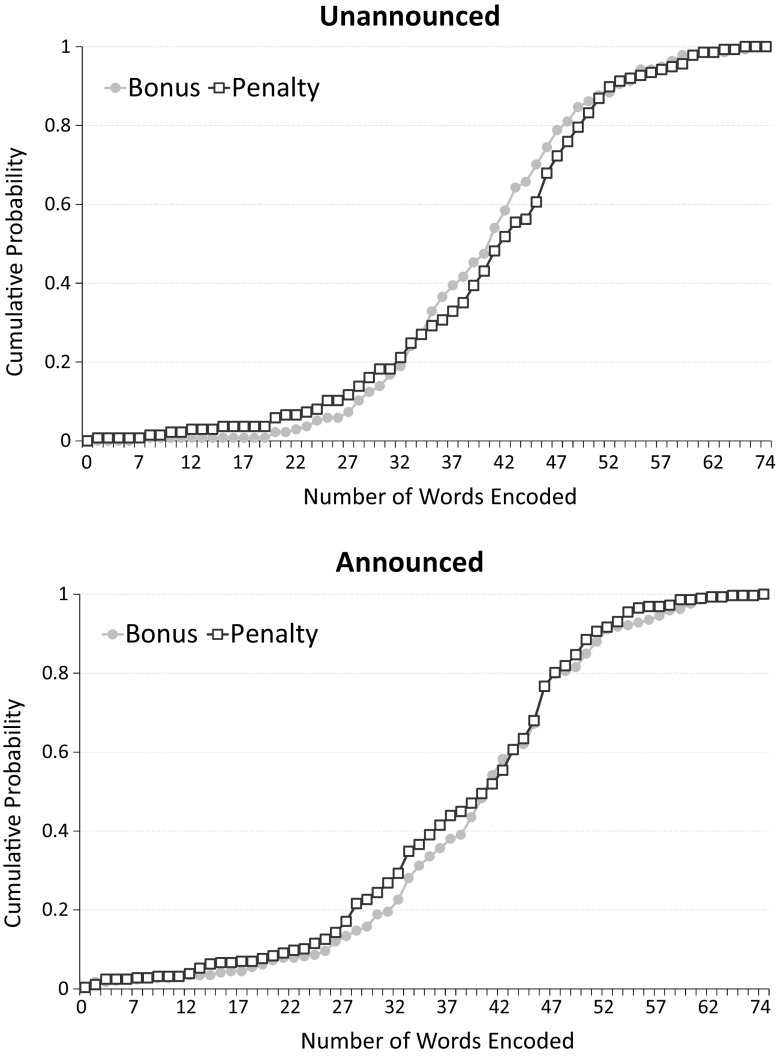
Performance across treatments

Figure 2 shows the cumulative distribution functions (CDFs) of the numbers of words encoded by participants in Part 2 of the experiment. The top and bottom panels show the CDFs of the Bonus and Penalty treatments for the Unannounced and Announced conditions, respectively.

First of all, note that in Announced we observe about 23% of subjects encoding more than the target of 45 words. There are three possible explanations for this result: one is that, in addition to extrinsic incentives, workers are intrinsically motivated to provide effort, perhaps because they enjoy the task. Another possibility is that subjects care about reputation on top of monetary incentives. A third possibility is a gift-exchange hypothesis: since workers are always being paid something, they respond by providing effort.^13^

Regarding contract framing effects, in both conditions the CDFs of Bonus and Penalty overlap substantially, indicating very small differences in performance. In Unannounced, subjects encoded on average 41 words (s.d. = 11.5) in Penalty and 40 words (s.d. = 9.78) in Bonus. The difference is statistically insignificant (*p* = 0.407 using a two-sided Mann–Whitney test; *p* = 0.513 using a two-sided Kolmogorov–Smirnov test; 137 observations per treatment). In Announced, subjects in Penalty encoded on average fewer words (38; s.d. = 12.4) than in Bonus (39; s.d. = 12.0). This difference is also insignificant (*p* = 0.291 using a two-sided Mann–Whitney test; *p* = 0.383 using a two-sided Kolmogorov–Smirnov test; 292 and 287 observations in Bonus and Penalty, respectively). Moreover, we find no difference between contract framing effects between Announced and Unannounced conditions.^14^

## Discussion and conclusion

In our experiment subjects perform a real-effort task and are paid for meeting a performance target. The incentives are framed either as “bonuses” or “penalties” for meeting/not meeting the target. We conducted two sets of experiments in which the target was either announced at the beginning of the task or not. In both settings, we find that performance is statistically indistinguishable between bonus and penalty frames. The absence of a contract framing effect when the target is announced is consistent with the findings of Brooks et al. (2012), DellaVigna and Pope (2016), and Grolleau et al. (2016). However, our finding that performance is unresponsive to framing when the target is unannounced contrasts with results reported by Hannan et al. (2005), Armantier and Boly (2015), and Imas et al. (2017).

What can explain our failure to replicate a contract framing effect when the target is unannounced? First of all, we emphasize that, given the average effect size observed in the literature (about 0.5), our study is highly powered, and so the null result is not due to a lack of power to detect an effect of such size. Thus, one way to interpret our results is that there is no effect of framing on effort provision. However, this conclusion is conditional on the true magnitude of the effect being as large as reported in previous studies. Moreover, this leaves unexplained why several previous studies did find significant framing effects.

We believe that a more plausible interpretation of our results is that the “true” effect of contract framing is simply smaller than previously reported. We can conduct a meta-analysis of the effect sizes observed in the literature to get a more precise estimate of the true effect. Using the effect sizes and standard errors reported by Hannan et al., Armantier and Boly and Imas et al., as well as our own Unannounced treatments, we can compute a weighted mean estimate of the effect size equal to 0.313.^15^ We can repeat the analysis for the Announced condition, combining our data with that of Brooks et al. DellaVigna and Pope and Grolleau et al. The weighted mean estimate of the effect size is 0.003. Finally, we can compute an estimate of the effect size combining the two conditions and using the data from all studies reported in Fig. 1 as well as our treatments. This is equal to 0.071.

Finally, a word of caution should be spent about the specific subject pool used in our study, MTurk workers. One may worry that the small effect of framing in our study is due to the fact that MTurkers are generally unresponsive to the type of (small) monetary incentives used in experiments (e.g., because they mainly care about reputation). However, as discussed above, this is unlikely to be the case: the pay-per-performance incentives used in the experiment raise effort substantially relative to a control treatment with flat payments (see footnote 11 and Appendix C in electronic supplementary material). Nevertheless, there is some evidence that interventions that rely on subtle psychological manipulations, such as contract framing, may produce somewhat weak effects in this setting: DellaVigna and Pope (2016), for example, find limited evidence of contract framing effects as well as of probability weighting on a large sample of MTurkers. Similarly, while the experiment conducted by de Quidt (2017) on MTurk identifies a significant contract framing effect, the reported effect size is smaller (about 0.2) than those reported in previous lab experiments.^16^ While our study cannot draw definitive conclusions about the role of subject pool idiosyncrasies, this seems an important question for future research.

## Electronic supplementary material

Below is the link to the electronic supplementary material.

Supplementary material 1 (DOCX 2.07 MB)

## Footnotes

1 Armantier and Boly (2015) ran one experiment in Burkina Faso and one in Canada, and therefore we report two effect sizes. We do not include de Quidt (de Quidt 2017) in Fig. 1 because his subjects were allowed to self-select into treatment.

2 Hedges’ *g* is similar to Cohen’s *d* with a correction for small samples. It is measured as $g = \left( {\mu_{p} - \mu_{b} } \right)/\sqrt {\left[ {\left( {n_{p} - 1} \right){\text{sd}}_{p}^{2} + \left( {n_{b} - 1} \right){\text{sd}}_{b}^{2} } \right]/\left( {n_{p} + n_{b} - 2} \right)}$ where $\mu_{p}$ and $\mu_{b}$ are the mean efforts in the penalty and bonus treatments, ${\text{sd}}_{p}$ and ${\text{sd}}_{\text{b}}$ their respective standard deviation and $n_{p}$ and $n_{b}$ the sample sizes. Thus, Hedges’ *g* measures the standardized difference in effort due to the framing of the incentive scheme. The studies shown in Fig. 1 vary, however, in the level of incentives (i.e. the ratio between base salary and bonus/penalty) offered to subjects, and one should keep this in mind when comparing effect sizes across Fig. 1 since the effect of framing might vary with the incentive level (Armantier and Boly 2015).

3 In DellaVigna and Pope (2016) subjects participated in a real-effort task and received a bonus/penalty based on a performance target that was specified ex-ante. Brooks et al. (2012) conducted a chosen-effort experiment where subjects knew in advance whether any level of effort resulted in a bonus/penalty. Grolleau et al. (2016) gave subjects pairs of matrices containing numbers and, in each pair, subjects had to find two numbers that added up to ten. They were paid a piece-rate for each correctly solved pair of matrices.

4 In Imas et al. (2017) incentives were contingent upon meeting a target that was not specified ex-ante. In Hannan et al. (2005) effort only affected the probability of meeting the target. In Armantier and Boly (2015) participants were recruited to spell-check exam papers and bonuses/penalties depended on the quality of their spell-checking (verified ex-post by the experimenters).

5 MTurk is an online labor market. The experiments were conducted using the software LIONESS (Aréchar et al. 2017). Subjects were adult residents of the US. See Horton et al. (2011) for a discussion of experiments conducted on MTurk.

6 Subjects had to encode a word correctly before they could proceed to the next. The letter-to-number assignments were kept constant across the whole experiment. See Appendix B in electronic supplementary material for the experimental instructions.

7 We set the target at 45 words based on the results of a pilot conducted to calibrate incentives.

8 This is similar to Imas et al. (2017). The target was based on the average performance of subjects in the Announced condition and equal to 39 words. Note that this might induce an additional effort motive in Unannounced: because there is an implicit comparison to the performance of others, some workers may respond differentially to penalties because they expect that *other* workers will. Also note that the target is lower in Unannounced than in Announced. Because the true value of the target was announced only at the end of the experiment, this should not affect performance.

9 In Part 3 subjects were initially given $1.20 and losses were subtracted from this initial payment. At the end of the experiment, if Part 3 was selected for payment, one lottery was chosen at random and, if accepted, played out to determine final payments.

10 This belief elicitation was not incentivized. The average belief was 32 in Bonus (s.d. = 12.8) and 31 in Penalty (s.d. = 12.1), and the difference is insignificant (*p* = 0.899 using a two-sided Mann–Whitney test; *p* = 0.769 using a two-sided Kolmogorov–Smirnov test; 137 observations per treatment).

11 We conducted one additional treatment (*N* = 140) where in Part 2 subjects received a flat payment of $0.50, regardless of how many words they encoded. With this control treatment we assess whether subjects are responsive to the type of incentives used in the Bonus/Penalty treatments. (We thank an anonymous referee for suggesting this treatment). This could be important since MTurkers may be partly motivated by reputation (e.g., their work needs to be “approved” by the employer before a payment can be made and the approval rate is a statistic that future employers can use to screen workers). Reputational concerns may dilute the effect of short-term monetary incentives offered in the experiment, making it harder to find framing effects. We find large differences in effort between the control and Bonus/Penalty treatments (see Appendix C in electronic supplementary material), suggesting that MTurkers are responsive to the type of monetary incentives offered in our task. See also DellaVigna and Pope (2016) fur further corroborating evidence.

12 Given the average effect size of 0.08 reported across the three studies with announced targets, the achieved power in the Announced condition is low (16%). An 80% power could only be achieved with a sample of about 2450 subjects per treatment. This is due to the very small effect sizes reported in DellaVigna and Pope (−0.07) and Grolleau et al. (0.03). The effect size reported in Brooks et al. (0.26) can be detected given our sample size.

13 However, as shown by our control experiment reported in Appendix C in electronic supplementary material, all these additional motives do not seem to offset the effectiveness of monetary incentives.

14 See Appendix D in electronic supplementary material where we report a pooled regression with dummies for Penalty, Unannounced, and their interaction. The coefficient of the interaction term is very small (0.13) and insignificant (*p* = 0.922). In electronic supplementary material (Appendix D) we also report an analysis of the interaction of framing effects and degree of loss aversion, as measured in the lottery task of Part 3 of the experiment. We find no evidence of a contract framing effect even among the most loss averse participants.

15 This is computed as $\mathop \sum \nolimits_{i = 1}^{k} w_{i} g_{i} /\mathop \sum \nolimits_{i = 1}^{k} w_{i}$ where $g_{i}$ is the effect size of study *i* and $w_{i}$ is its weight, equal to the inverse of the within-study variance.

16 However, in his study subjects self-selected into treatment, which may affect the estimate of the effect size.
