# Supplementary material for: Bonus versus penalty: How robust are the effects of contract framing?
Source: J Econ Sci Assoc. 2017 Sep 23;3(2):174–82. doi: 10.1007/s40881-017-0039-9 (PMC6956932; doi:10.1007/s40881-017-0039-9)
Supplement: Supplementary file 1 — Supplementary material 1 (DOCX 2.07 MB) [file 40881_2017_39_MOESM1_ESM.docx]

**Supplemental Material**

**Appendix A: Details of studies included in Figure 1**

Table A1 shows details of the experimental designs of previous studies on incentives framing included in Figure 1 in the main text.

**Table A1 – List of studies included in Figure 1 in the main text**

| **Study** | **Country** | **Subject pool** | **N** | **Task** | **Incentive scheme** | **Target announced?** |
| --- | --- | --- | --- | --- | --- | --- |
| Armantier & Boly (2015)* | Burkina Faso | Students and general population with a degree | B = 29  P = 34 | Grading exams | Base pay: FCFA500.  B/P: three levels FCFA  1500/2500/4500, depending on the “grading quality”, i.e. the proportion of mistakes correctly identified. | No |
| Hannan et al. (2005) | United States | M.B.A. students | B = 35  P = 33 | Chosen effort | Base pay: $20  B/P: $10 | No |
| Imas et al. (2017) | United States | Students | B = 40  P = 43 | Slider task | Base pay: none  B/P: t-shirt (cost $9) | No |
| Armantier & Boly (2015)* | Canada | Students | B = 58  P = 56 | Grading exams | Base pay: C$2.08  B/P: three levels, depending on target, C$6.25/10.42/18.75 | No |
| Brooks et al. (2012) | Switzerland | Students | B = 72  P = 73 | Chosen effort | Base pay: CHF 20  B/P: CHF 5 | Yes |
| DellaVigna and Pope (2016) | United States | MTurkers | B = 545  P = 532 | Pressing a-b keys | Base pay: $1  B/P: $0.4 | Yes |
| Grolleau et al. (2016) | France | Students | B = 150  P = 150 | Finding two numbers that add up to 10 in pairs of matrices | Base pay: none  B/P: €1.5 per pair of matrices solved/unsolved | Yes |
| *Notes*: B = Bonus; P = Penalty; FCFA = CFA Franc; $ = US Dollar; C$ = Canadian Dollar; CHF = Swiss Franc; € = Euro  * Armantier & Boly (2015) also conduct a treatment with both bonus and penalties, depending on performance (N=34 in Burkina Faso and N=56 in Canada); | | | | | | |

**Appendix B: Experimental instructions**

**[Common to all treatments]**

Introduction


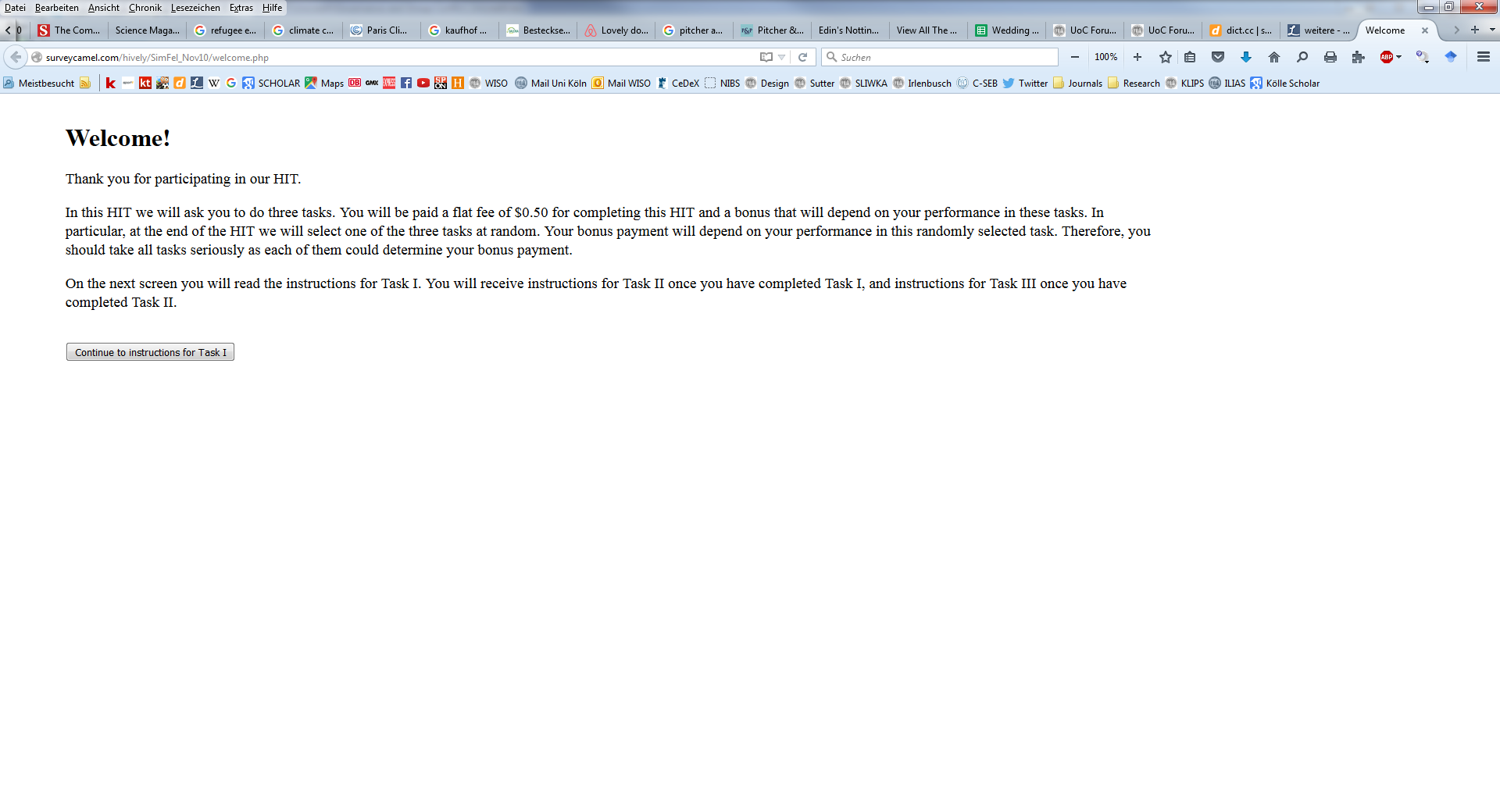


Part 1 instructions


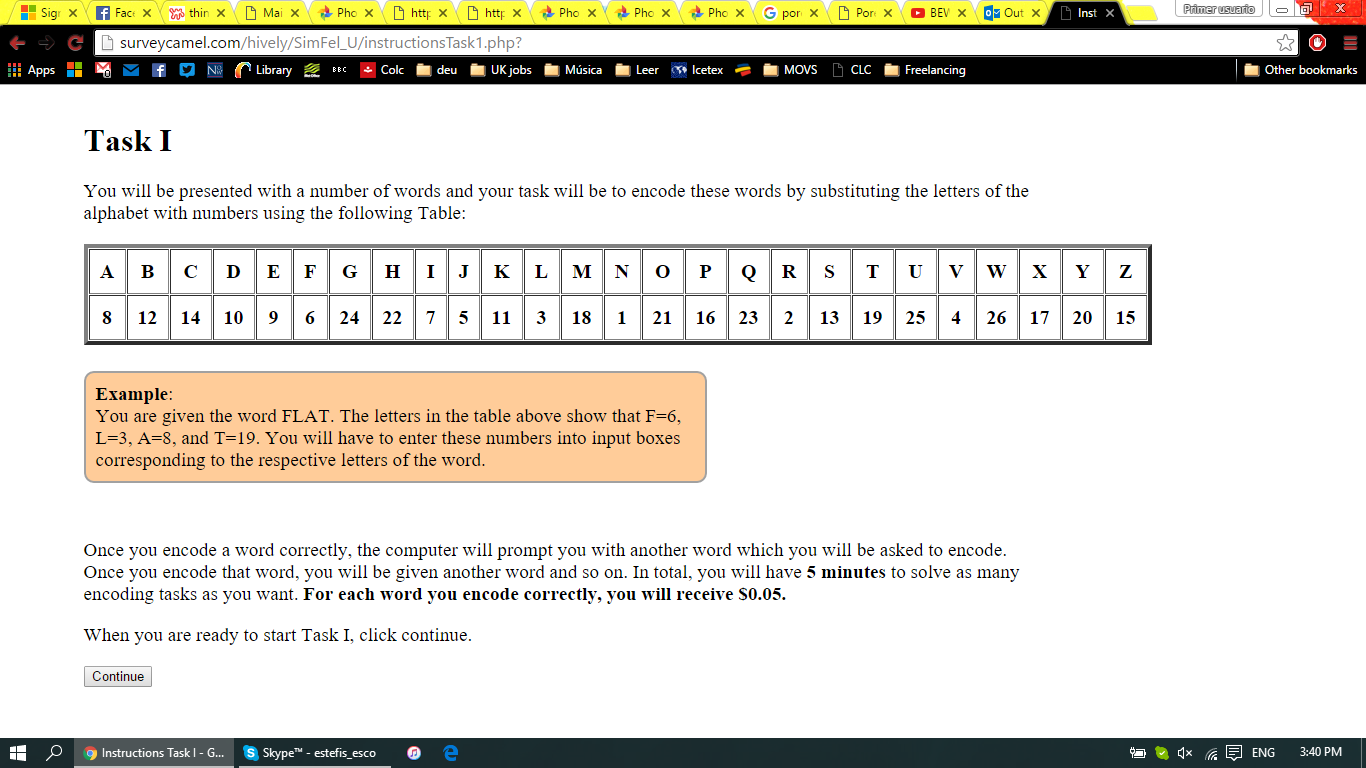


Part 1 task


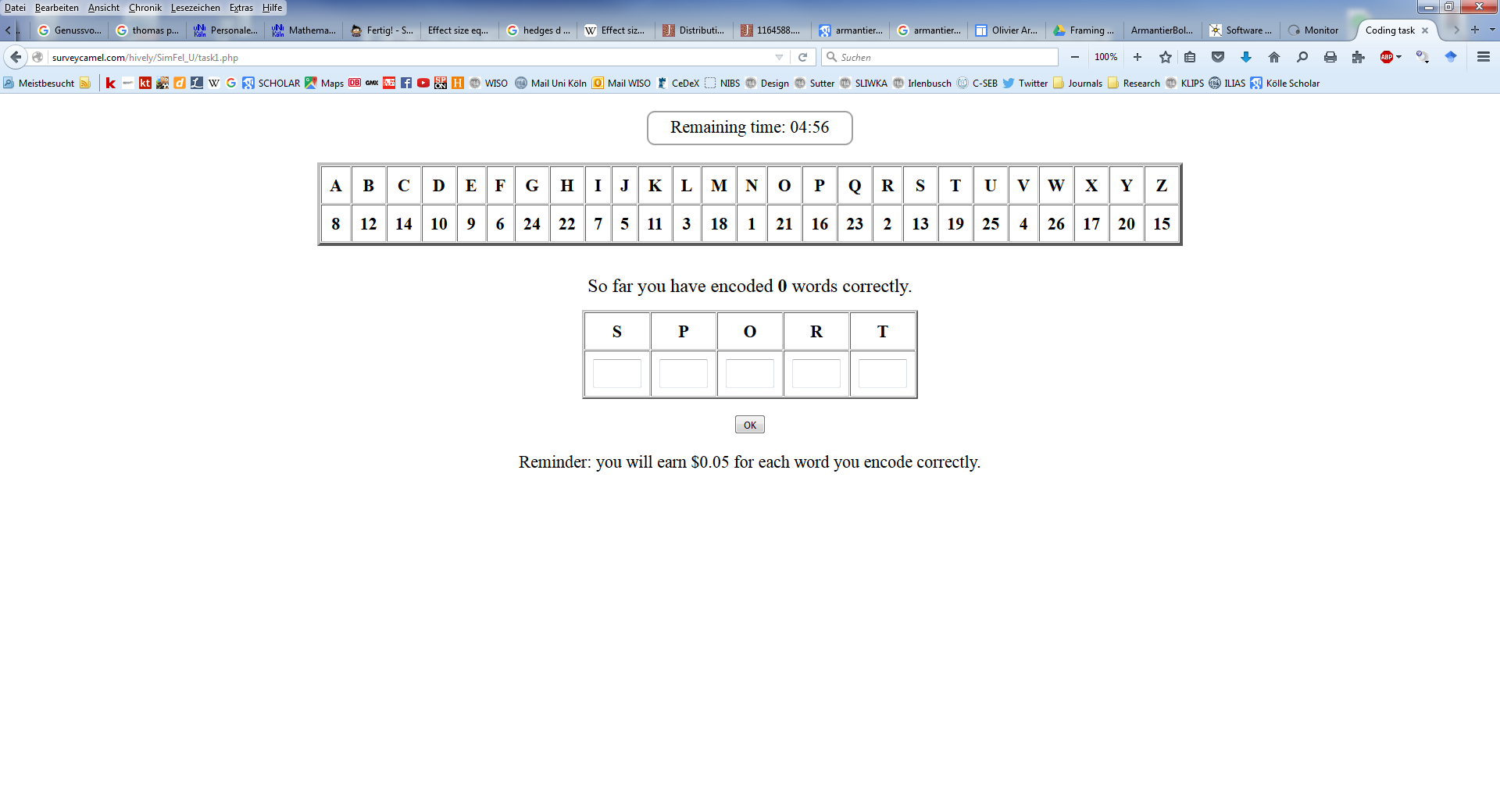


Part 1 feedback


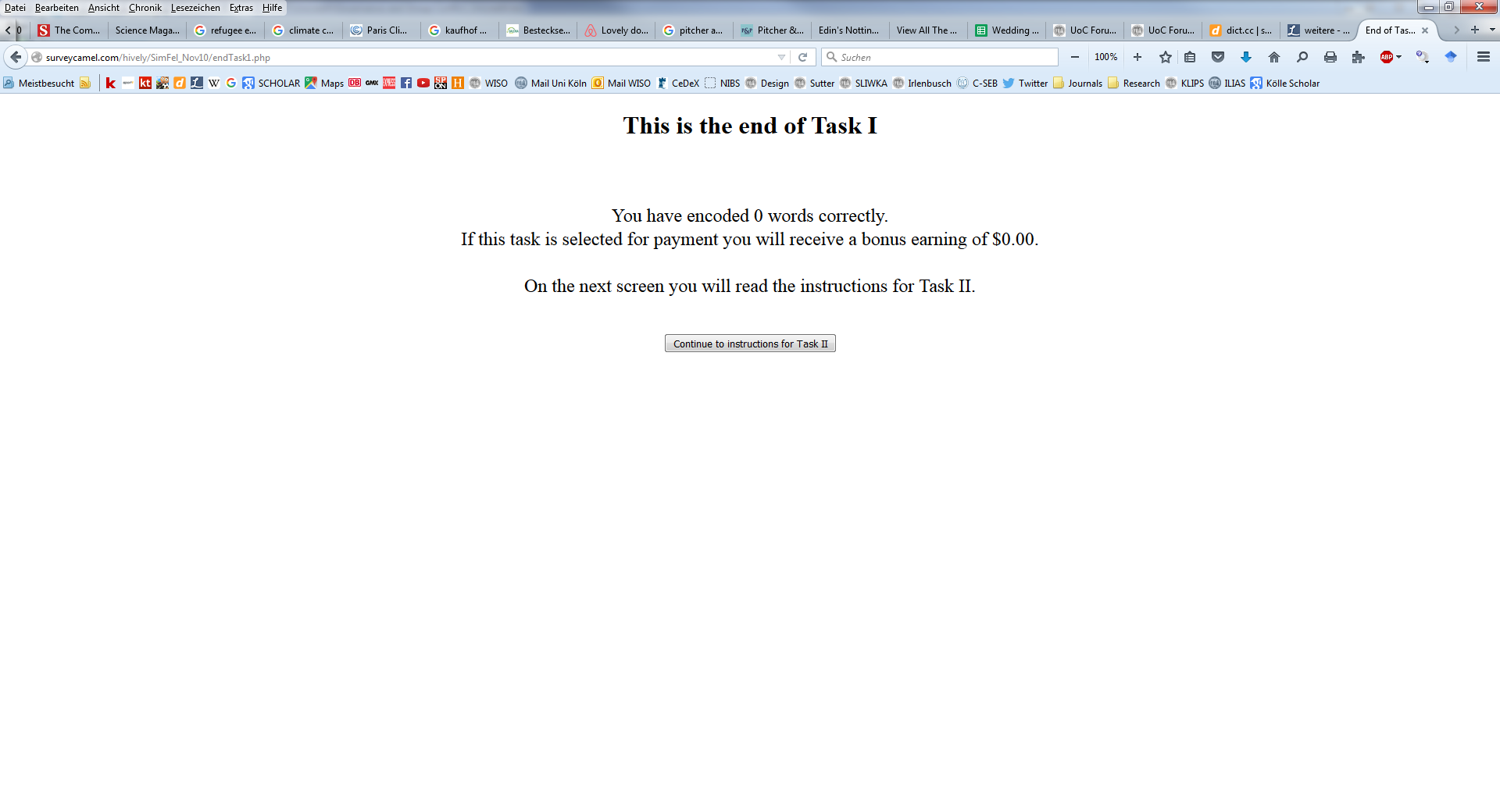


**[Unannounced Fine]**

Part 2 instructions


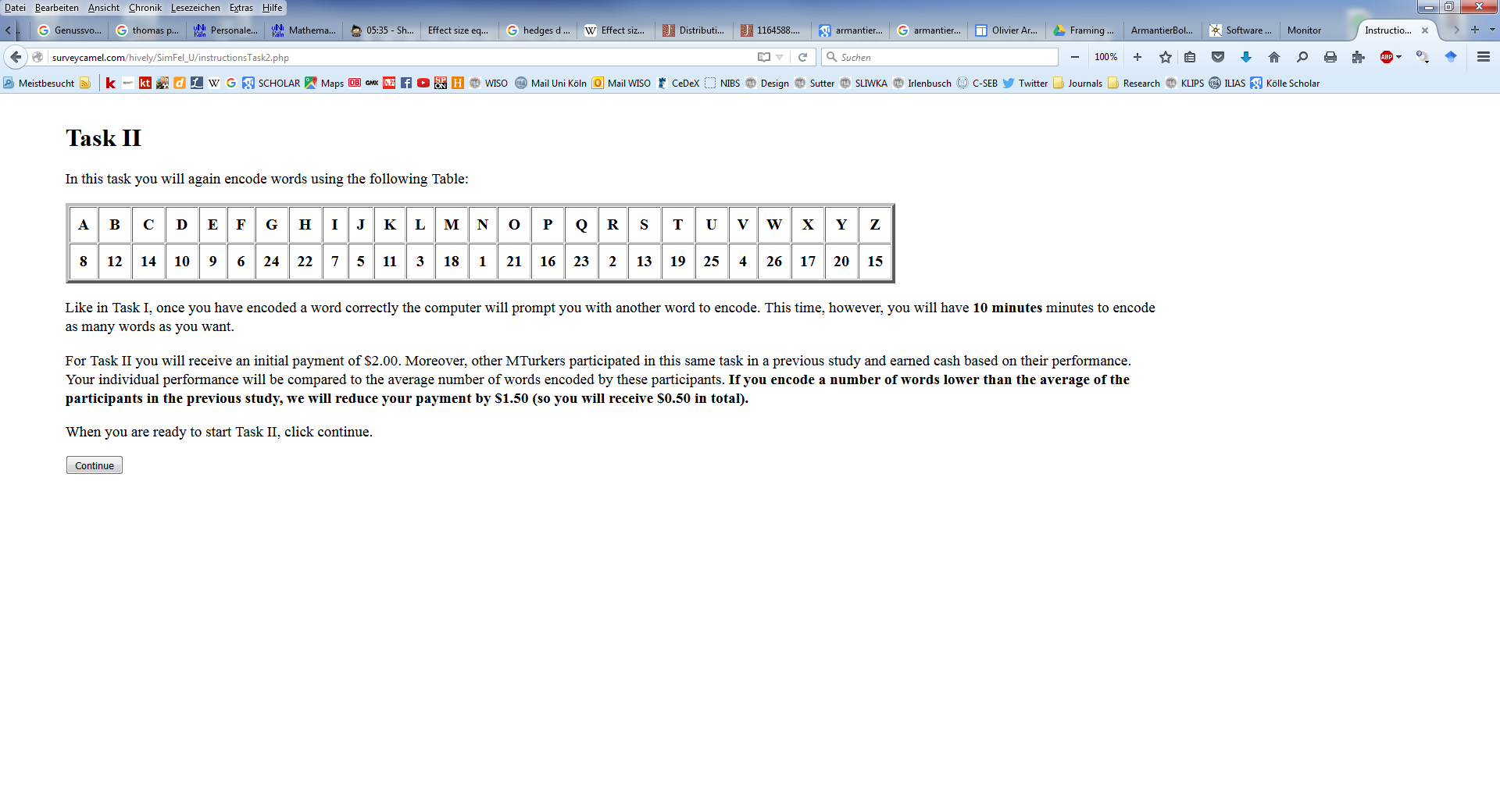


Part 2 task


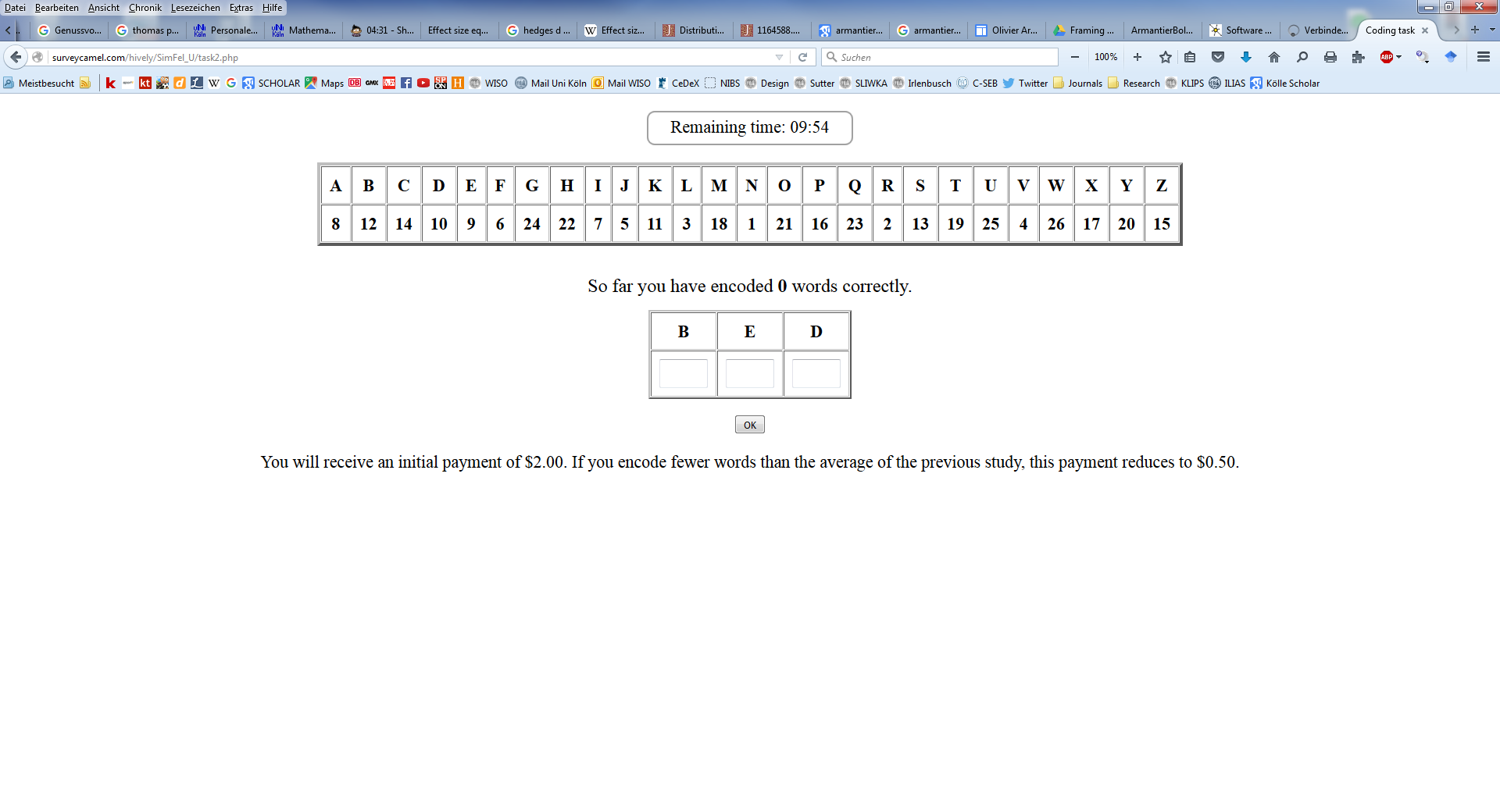


Part 2 belief


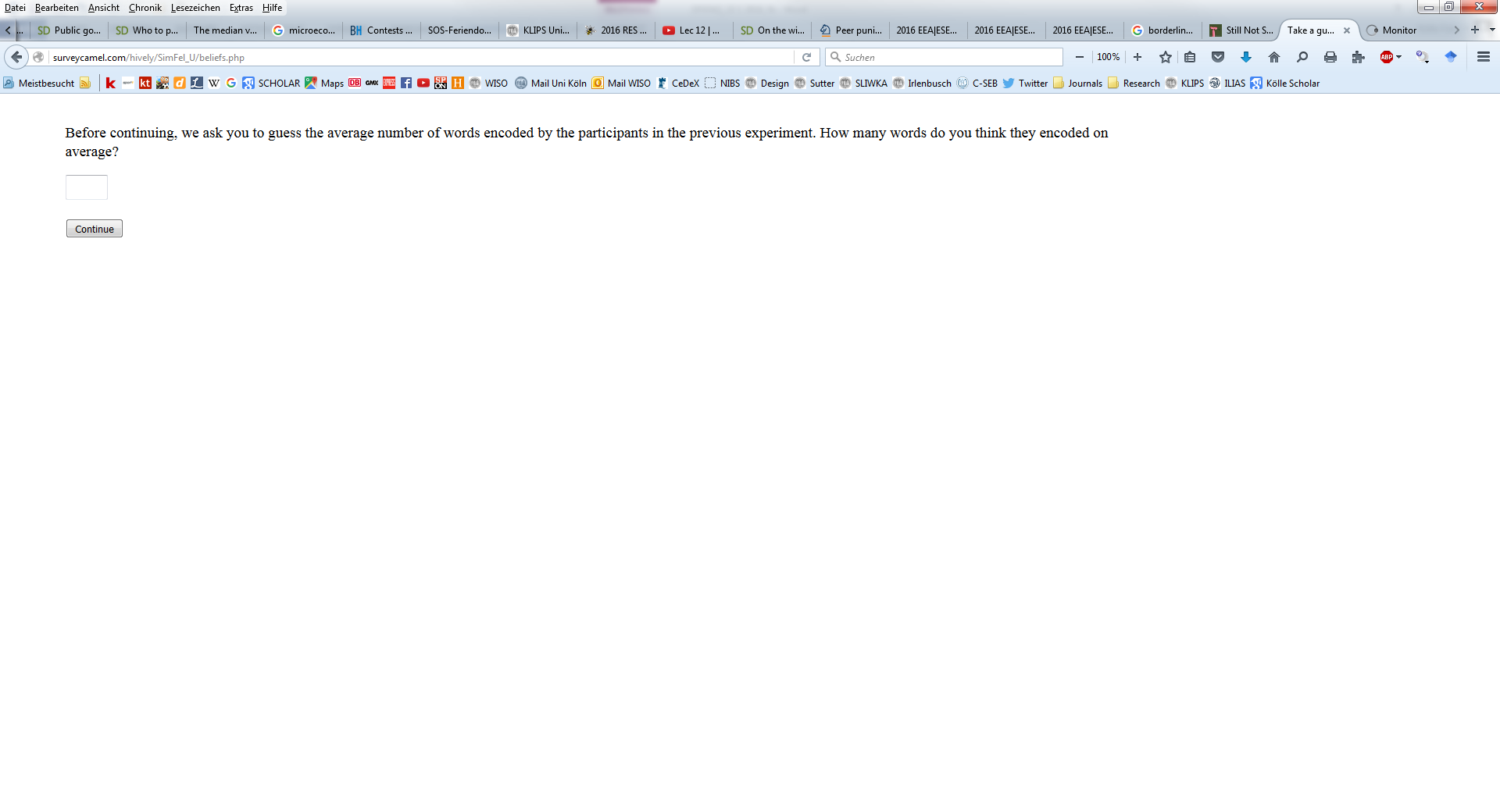


**[Announced Fine]**

Part 2 instructions


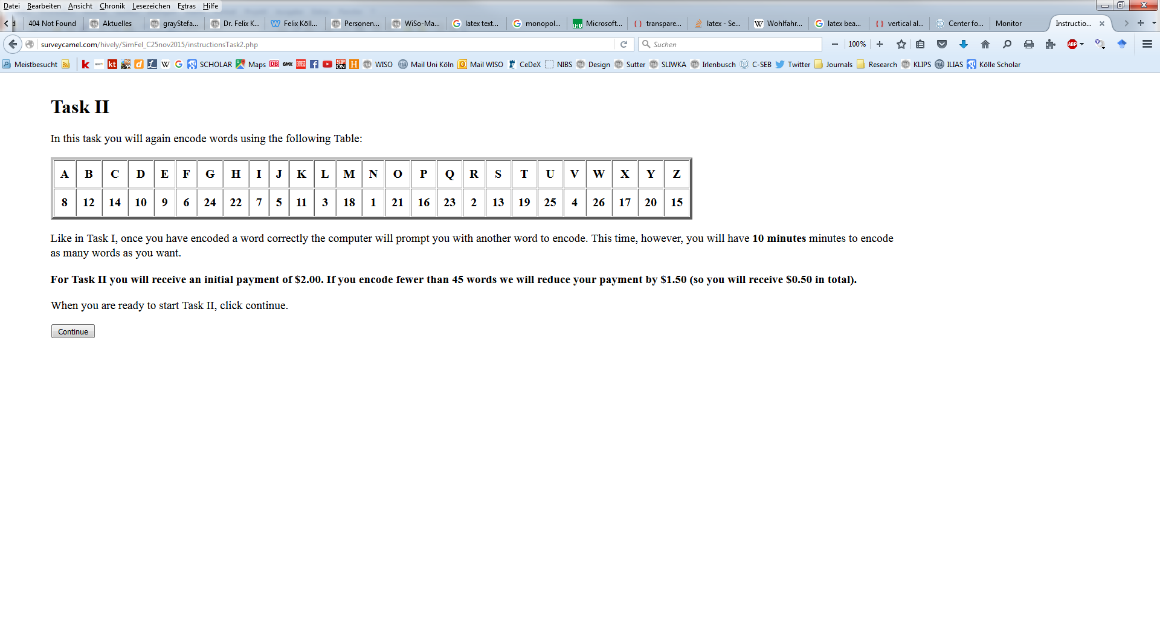


Part 2 task


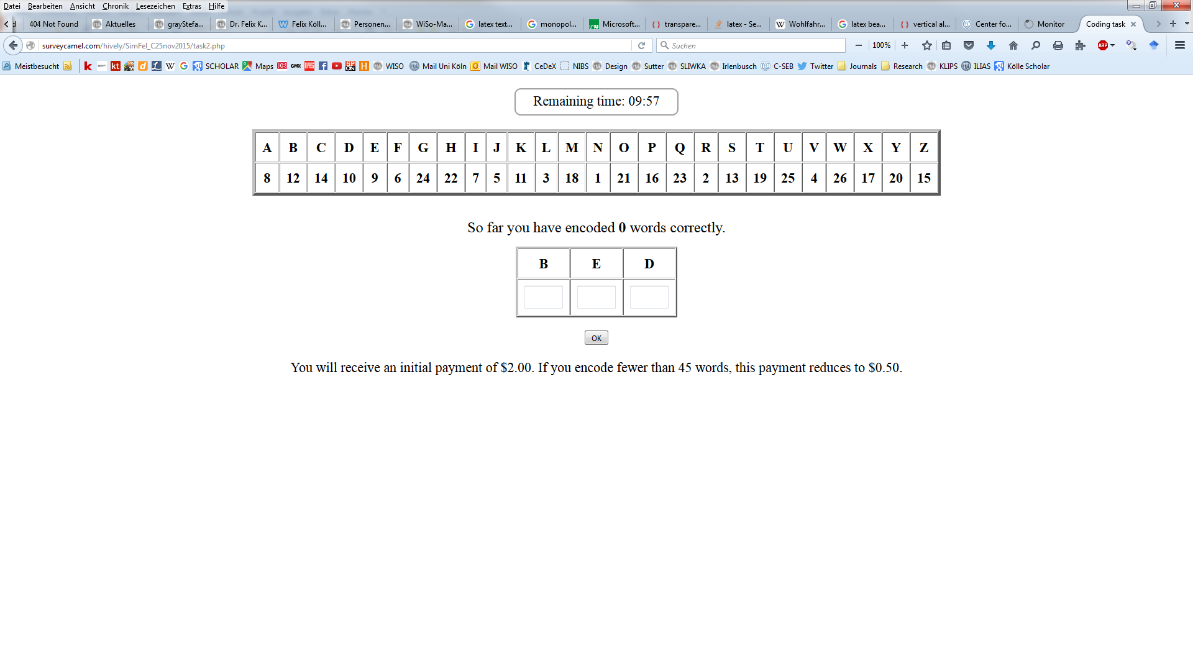


**[Unannounced Bonus]**

Part 2 instructions


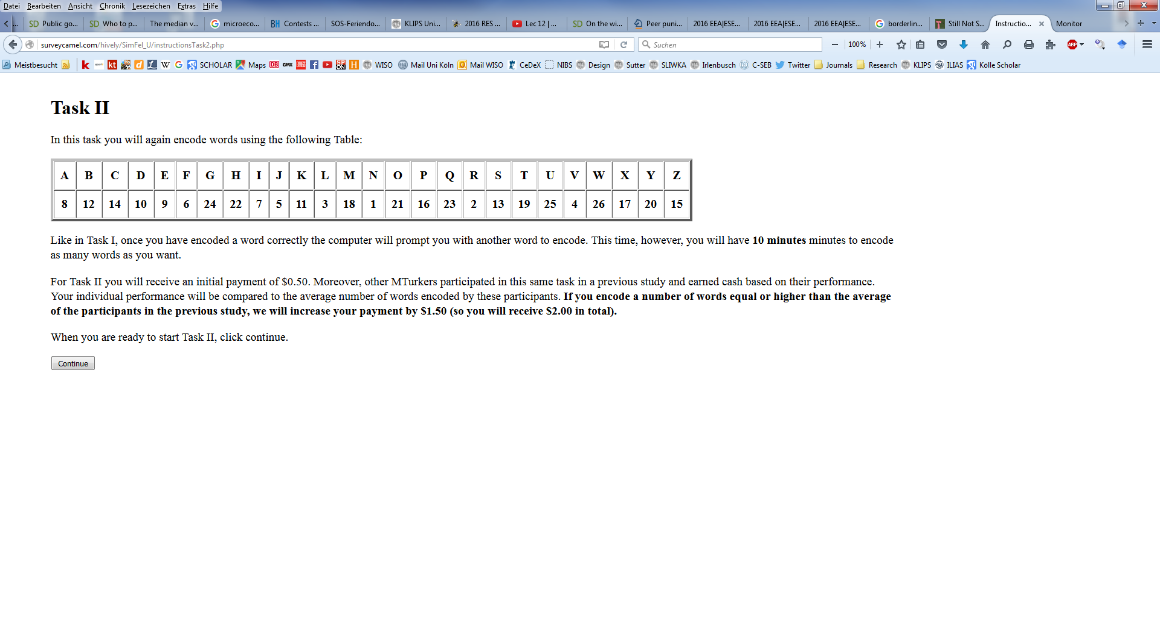


Part 2 task


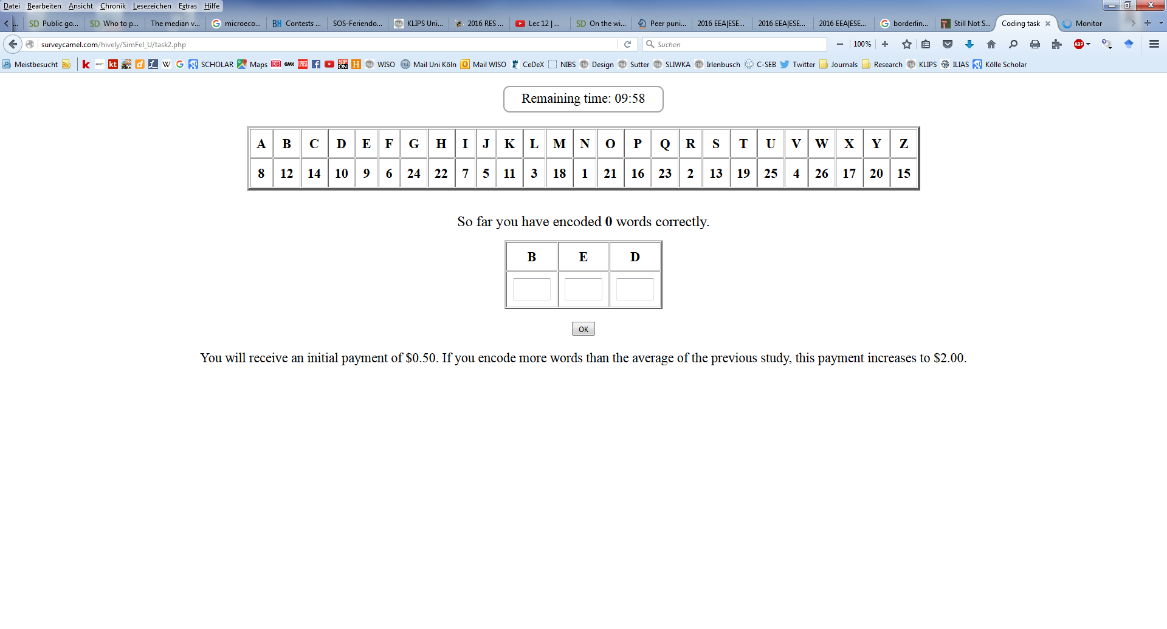


Part 2 belief


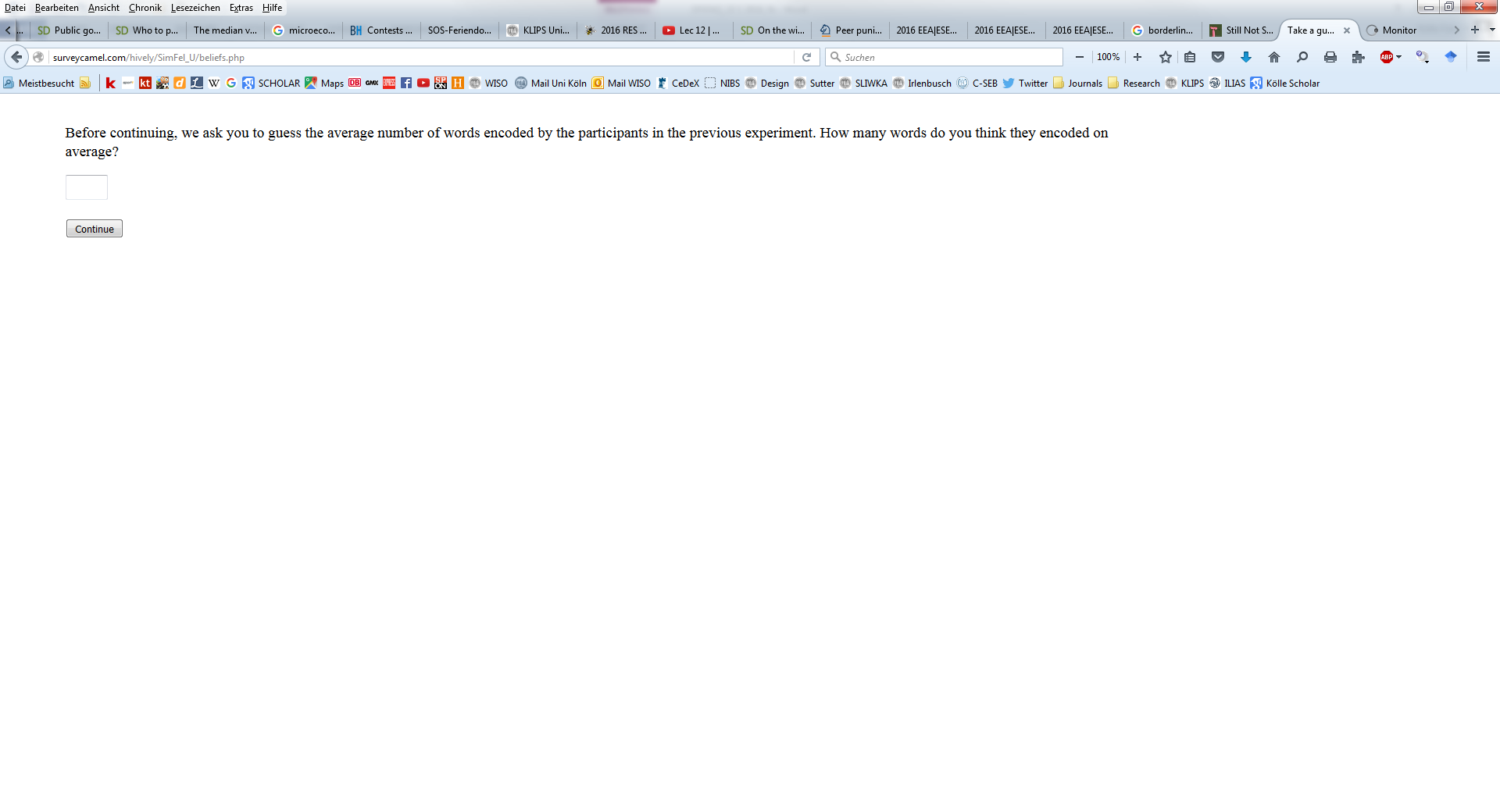


**[Announced Bonus]**

Part 2 instructions


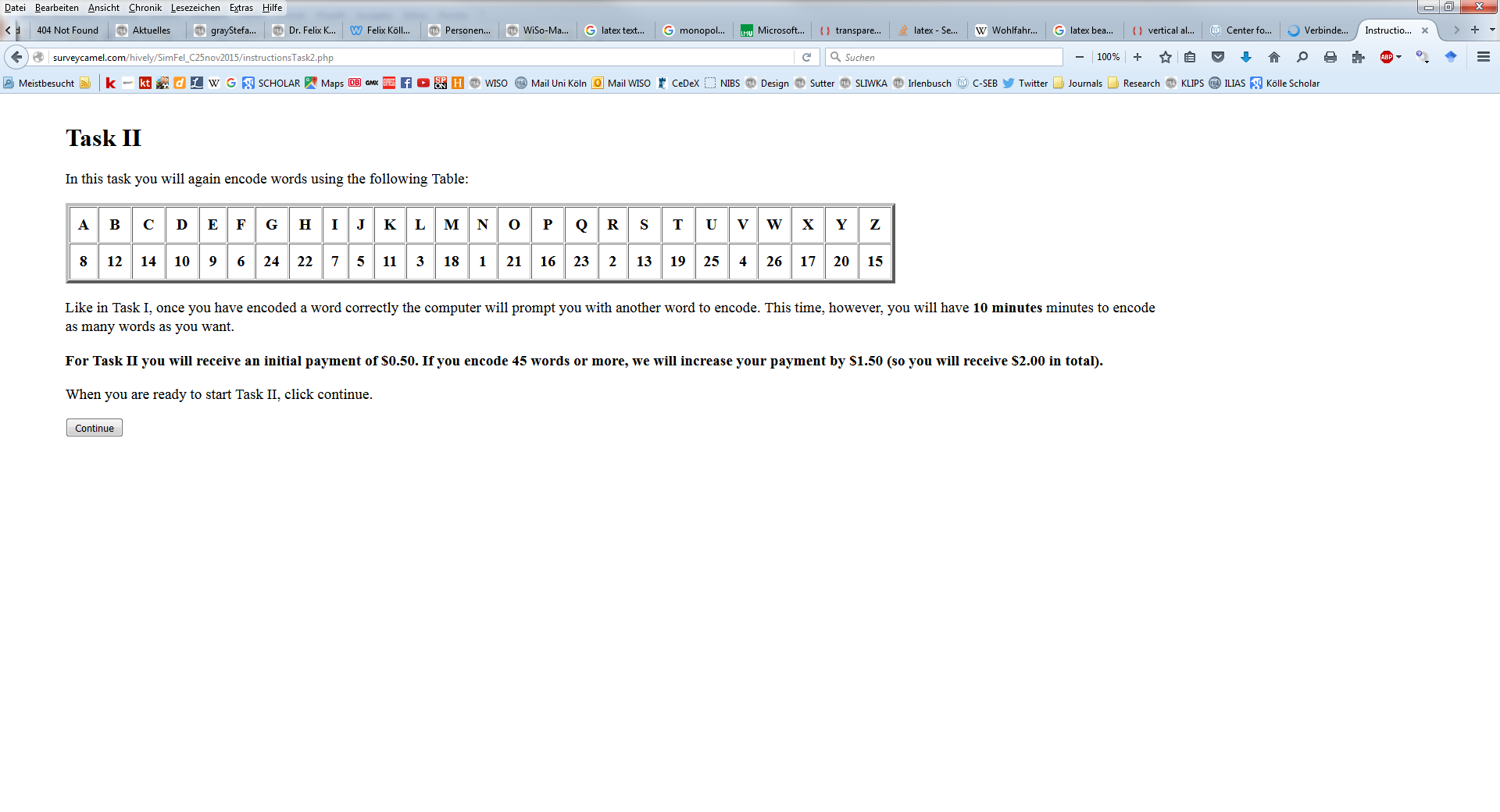


Part 2 task


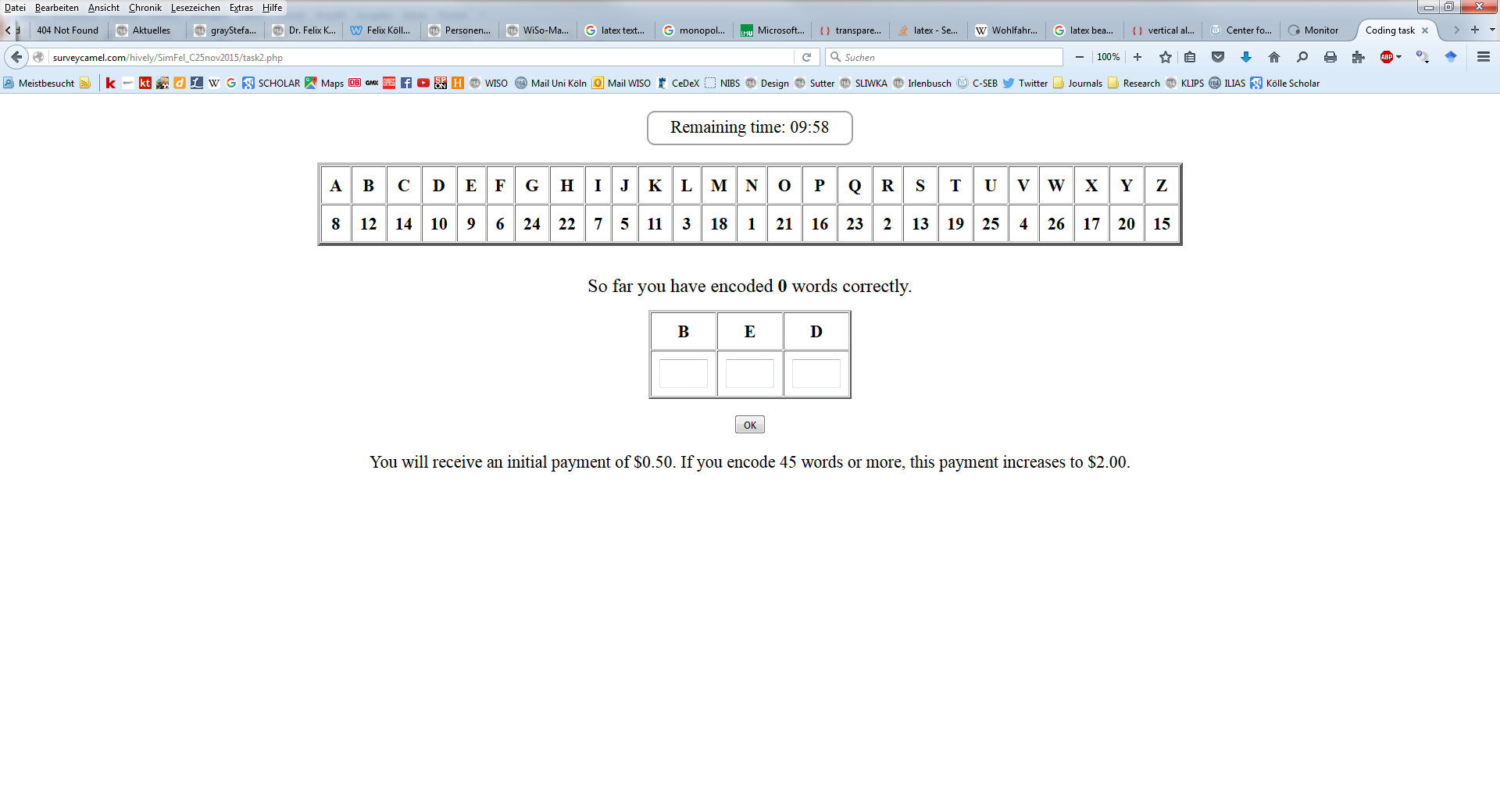


**[Common to all treatments]**

Part 3


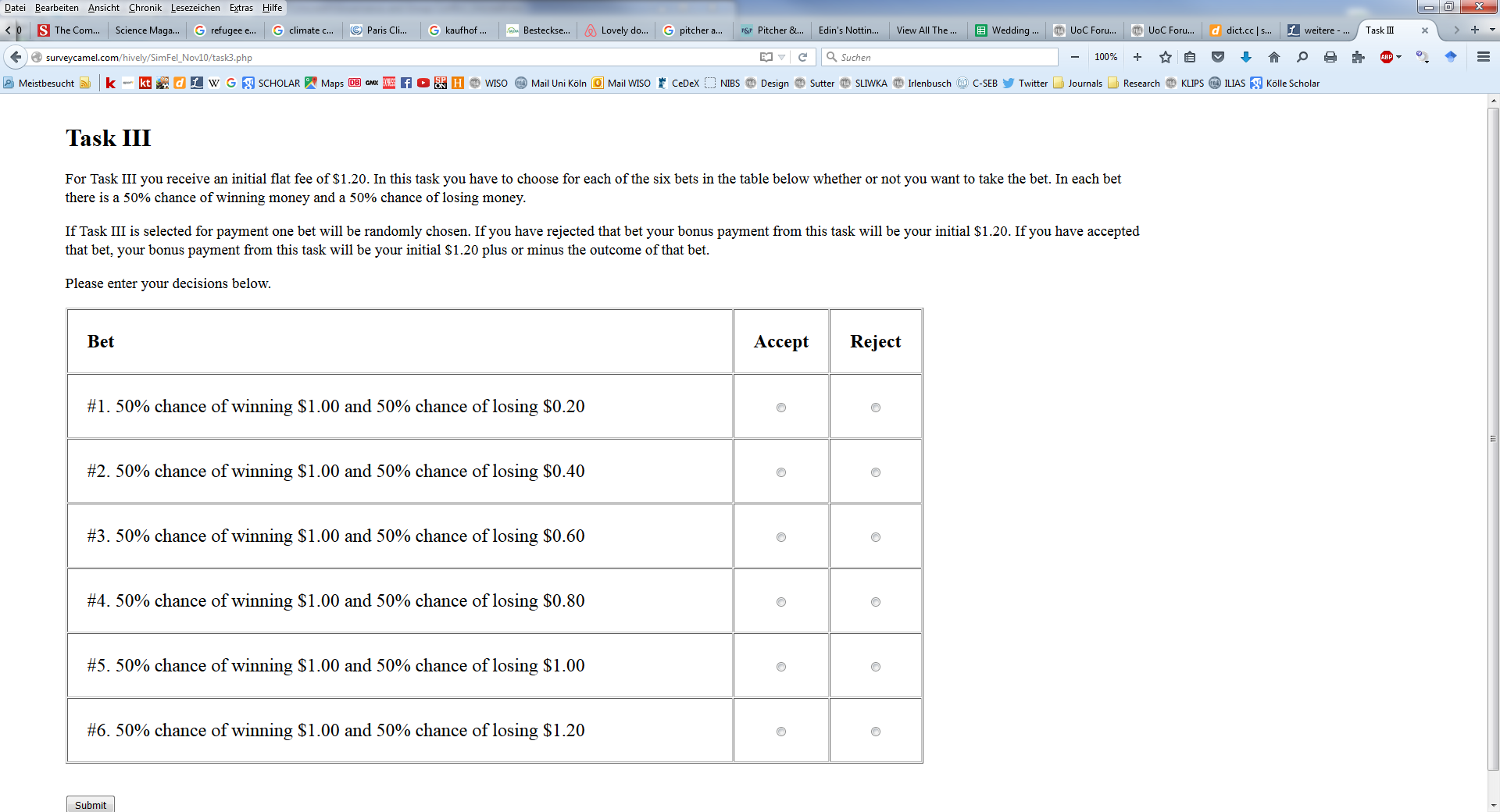


**Appendix C: Analysis of Control Treatment**

To test whether subjects in our subject pool react at all to short-term monetary incentives, we conducted an additional treatment (**Flat**) with N = 140 participants. This treatment is identical to our other treatments except for the fact that in Part 2 there was no bonus or penalty for (not) meeting a performance target. Instead, subjects received a flat payment of $0.50, regardless of the number of words encoded correctly.

If effort is costly and if subjects are solely motivated by maximizing their own income, then we should observe zero effort in Part 2 of this treatment. If subjects exhibit intrinsic motivation for providing effort (e.g. because they enjoy the task) or if they exhibit some form of other-regarding preferences towards the experimenter (e.g. gift-exchange hypothesis), or other types of reputational considerations (e.g. they worry that if they do not work the experimenter will rate them negatively at the end of the study), then positive effort levels are possible. Yet, we expect these effects to be weaker than the monetary incentives we provided in our Bonus and Penalty treatments. We thus predict that performance, measured as the number of encoded words in Part 2, is lower in our Flat treatment compared to our Bonus and Penalty treatments. We expect no such differences for Part 1 as this was identical for all treatments (subjects always received a piece-rate for words encoded correctly).

These hypotheses are supported by the data. Table C1 compares the work performance for Part 1 and Part 2 across the Flat treatment and our Bonus and Penalty treatments. As we found no differences across Bonus and Penalty, in the following we pool data from these treatments. We also pool across our Announced/Unannounced conditions, but the results hold if we compare Flat separately with each treatment.

As expected, in Part 1 performance is very similar and statistically indistinguishable across treatments. This is further illustrated in Figure C1, showing a substantial overlapping of the cumulative distribution functions (CDFs). This is important because it shows that workers recruited for the Flat and Bonus/Penalty treatments are not different per se in terms of productivity.

In Part 2, in contrast, we find large differences between Flat and the Bonus/Penalty treatments. While in the former participants encode on average around 25 words, in Bonus/Penalty they encode on average 39 words. This difference is highly significant both according to a Mann-Whitney U test as well as a Kolmogorov-Smirnov test. Figure C2 corroborates this finding, showing a stark shift in the cumulative distribution function. In Flat, it further reveals that while there are some participants who indeed put in very little effort (24% of the participants encoded less than 10 words compared to 2% in the Bonus and Penalty treatment), a majority of subjects put in a positive amount of effort even in the absence of monetary incentives. As explained above, this observation is well in line with previous evidence showing that many people are not solely motivated be pecuniary incentives but also by, e.g., intrinsic motivations or other-regarding preferences.

**Table C1 – Performance across the Flat and the Bonus/Penalty treatments**

|  | **Bonus / Penalty treatments  (n = 853)** | **Flat treatment  (n = 140)** | **Mann-Whitney U test** | **Kolmogorov-Smirnov test** |
| --- | --- | --- | --- | --- |
| **Num. words encoded in Part 1** | 17.45  (5.62) | 17.84  (4.77) | *p* = 0.289 | *p* = 0.795 |
| **Num. words encoded in Part 2** | 39.33  (11.75) | 24.58  (16.33) | *p* < 0.001 | *p* < 0.001 |

*Note*: The table shows the average number of words encoded correctly. Standard deviation are in parentheses.

**Figure C1 – Performance across treatments in Part 1**

**Figure C2 – Performance across treatments in Part 2**

**Appendix D: Additional Analyses**

*D.1 Regression analysis of contract framing effects*

Table D1 reports an OLS regression of performance in Part 2 of the experiment for the Unannounced and Announced conditions. In both conditions, we regress performance (measured as number of words encoded in Part 2) on a treatment dummy Penalty (equal to 1 for subjects in the Penalty treatment), on a treatment dummy Unannounced (equal to 1 for subjects in the Unannounced treatment), an interaction term between the Penalty and Unannounced dummies, a control of individual ability (measured as number of words encoded in Part 1), a gender dummy, age, and a self-assessment of risk attitudes (measured using the SOEP general risk question).

**Table D1 – OLS regressions of performance across treatments**

|  | (1) |
| --- | --- |
|  |  |
| Penalty | -0.06  (0.72) |
| Unannounced | 1.56^*^  (0.91) |
| Penalty x Unannounced | 0.13  (1.28) |
| Num. Words in Part 1 | 1.47^***^  (0.06) |
| 1 if Female | 0.33  (0.61) |
| Age | -0.06^**^  (0.03) |
| Risk Loving | -0.05  (0.11) |
| Constant | 15.15^***^  (1.83) |
| R^2^ | 0.512 |
| N | 744 |

*Note*: Dependent variable is the number of words encoded in Part 2 of the experiment. For 4 subjects we have missing data on some questionnaire measurements and so they drop out of the regression analysis. Significance levels: ^***^ = 1%; ^**^ = 5%; ^*^ = 10%.

The regression confirms that the overall effect of contract framing is small and statistically insignificant in both the Unannounced and Announced conditions. Moreover, the insignificant interaction term indicates that there is no difference between framing effects in the Unannounced and Announced conditions. Among the controls, subjects’ ability in the encoding task is strongly and positively associated with performance in Part 2. Furthermore, older subjects tend to encode fewer words.

*D.2 Contract framing effects in sub-samples: loss aversion*

One common explanation for contract framing effects is loss aversion. In the following analysis we examine whether the effect of framing varies with subjects’ degree of loss aversion.

We start by reporting the distribution of loss aversion in our sample. We use the number of lotteries that a subject rejected in Part 3 of the experiment and compute his/her implied degree of loss aversion $\lambda$ (see Gächter et al. 2010 for details). Table D2 shows that a large fraction of subjects (63%) have $\lambda>2$, indicating substantial loss aversion in our sample. However, we also find heterogeneity, with 22% of subjects exhibiting a $\lambda$ close to (or smaller than) 1.

**Table D2 – Distribution of loss aversion in the sample**

| $\lambda$ | Unannounced | Announced | Overall |
| --- | --- | --- | --- |
| $>$ 5 | 10% | 8% | 8% |
| 5 | 22% | 28% | 26% |
| 2.5 | 31% | 27% | 29% |
| 1.67 | 15% | 15% | 15% |
| 1.25 | 8% | 11% | 10% |
| 1 | 5% | 6% | 6% |
| $\leq$ 0.83 | 9% | 5% | 6% |

*Note*: The computation of $\lambda$ is based on the number of lotteries rejected in Part 3, excluding subjects with multiple switch-points between accepting and rejecting lotteries (9% of subjects). The values of $\lambda$ are calibrated using the benchmark parameters in Gächter et al. (2010).

Figures D1 and D2 show the cumulative distribution functions (CDFs) of performance in Part 2 of the experiment in the Unannounced and Announced conditions, disaggregated by subject’s degree of loss aversion. In particular, we split our sample into two subgroups based on the median degree of loss aversion observed in the data ($\lambda=2.5$). The left panel of each Figure shows performance for the above-median subgroups, while the right panel shows the performance of the below-median subgroups.

**Figure D1 – Performance in Unannounced for below and above median loss averse subjects in Part 2**

**Figure D2 – Performance in Announced for below and above median loss averse subjects in Part 2**

For both subgroups, and both in Unannounced and Announced, the CDFs of Bonus and Penalty overlap substantially, indicating very small differences in performance. In Unannounced, below-median subjects in the Penalty treatment encoded on average 39.62 words (s.d. = 14.01) compared to 40.56 words (s.d. = 9.08) in Bonus. The difference is statistically insignificant (p = 0.741 using a two-sided Mann-Whitney test; p = 0.661 using a two-sided Kolmogorov-Smirnov test). In Announced, below-median subjects encoded on average 38.96 words (s.d. = 13.00) in the Penalty treatment and 40.27 words (s.d. = 13.46) in Bonus. This difference is also insignificant (p = 0.853 using a two-sided Mann-Whitney test; p = 0.616 using a two-sided Kolmogorov-Smirnov test).

Above-median subjects in Unannounced encoded on average 41.81 words (s.d. = 9.66) in Penalty and 41.14 words (s.d. = 10.37) in Bonus. The difference is insignificant (p = 0.556 using a two-sided Mann-Whitney test; p = 0.762 using a two-sided Kolmogorov-Smirnov test). In Announced, above-median subjects encoded on average 38.15 words (s.d. = 12.09) in Penalty and 39.27 words (s.d. = 11.27) in Bonus. The difference is also insignificant (p = 0.349 using a two-sided Mann-Whitney test; p = 0.337 using a two-sided Kolmogorov-Smirnov test).

Finally, in Table D3 we report OLS regressions of performance in Part 2 (measured as number of words encoded correctly) on a treatment dummy (1 if Penalty treatment), our measure of loss aversion (measured as the number of rejected lotteries in Part 3), and an interaction term between the treatment dummy and the loss aversion measure. We report separate regressions for the Announced and Unannounced treatments. In Unannounced, we also include subjects’ beliefs about the target as a regressor. Both regressions include additional controls for individual ability (number of words encoded in Part 1), gender, age, and risk attitudes (measured using the SOEP general risk question).

In both conditions we find that penalties have no impact among the least loss averse subjects (the Penalty dummy is insignificant in both Announced and Unannounced regressions). A subject’s degree of loss aversion has no impact on performance under bonus contracts, whereas under penalty contracts loss aversion has a positive effect on effort, and this is weakly significant in Unannounced. The direction of these effects is broadly consistent with the notion that loss aversion may play a role in explaining contract framing effects. However, the frame of the contract seems to have only a limited impact on performance in our setting.

**Table D3 – OLS regressions of performance**

|  | Unannounced treatments | Announced treatments |
| --- | --- | --- |
| Penalty | -2.86 | -2.94 |
|  | (1.90) | (2.00) |
|  |  |  |
| Loss Aversion | 0.03 | -0.24 |
|  | (0.29) | (0.35) |
|  |  |  |
| Penalty x Loss Aversion | 0.83^*^ | 0.78 |
|  | (0.48) | (0.50) |
|  |  |  |
| Belief about target | 0.22^***^ |  |
|  | (0.03) |  |
|  |  |  |
| Num. Words in Part 1 | 1.27^***^ | 1.47^***^ |
|  | (0.08) | (0.07) |
|  |  |  |
| Female | 1.40^*^ | -0.12 |
|  | (0.78) | (0.81) |
|  |  |  |
| Age | -0.06 | -0.07^*^ |
|  | (0.04) | (0.04) |
|  |  |  |
| Risk Loving | 0.21 | -0.07 |
|  | (0.14) | (0.15) |
|  |  |  |
| Constant | 11.12^***^ | 16.80^***^ |
|  | (2.60) | (2.76) |
| R^2^ | 0.715 | 0.464 |
| N | 243 | 531 |

*Note*: Dependent variable is number of words encoded in Part 2. Loss aversion is measured as the number of rejected lotteries in Part 3. Subjects with multiple switch-points in the lottery task are excluded. For 4 subjects we have missing data on some control variables and so they drop out of the regressions. Standard errors in parentheses. Significance levels: ^***^ = 1%; ^**^ = 5%; ^*^ = 10%.

*D.3 Contract framing effects in sub-samples: optimism*

It is also interesting to check whether the effect of contract framing in the Unannounced condition may depend on subjects’ beliefs about the target. Figure D3 shows the cumulative distribution functions (CDFs) of the numbers of performance in Part 2 of the experiment in the Unannounced condition, disaggregated depending on subjects’ beliefs about the target. In particular, we split our sample into two subgroups based on the median reported belief of the target (30 words). The left panel of each Figure shows performance for subjects who have “pessimistic” beliefs, i.e. subjects who believe that the target is relatively high, whereas the panel on the right shows performance for subjects who have relatively “optimistic” beliefs.

**Figure D3 – Performance in Unannounced for optimistic and pessimistic subjects with regard to the target in Part 2**

Subjects with pessimistic beliefs encoded on average 46.11 words (s.d. = 9.64) in Penalty and 45.49 words (s.d. = 8.53) in Bonus. The difference is insignificant (p = 0.331 using a two-sided Mann-Whitney test; p = 0.580 using a two-sided Kolmogorov-Smirnov test). Subjects with optimistic beliefs encoded on average 35.68 words (s.d. = 10.78) in Penalty and 36.64 words (s.d. = 8.94) in Bonus. The difference is also insignificant (p = 0.873 using a two-sided Mann-Whitney test; p = 0.965 using a two-sided Kolmogorov-Smirnov test).

Table D4 reports regression of number of words encoded in Part 2 among the two subgroups of subjects who hold pessimistic and optimistic beliefs about the target. The regressions include the usual controls. In both subgroups the penalty frame does not have a significant effect on performance.

**Table D4 – OLS regressions of performance for optimistic and pessimistic subjects with regard to the target in Unannounced**

|  | Unannounced treatment | |
| --- | --- | --- |
|  | Pessimistic | Optimistic |
| Penalty | 1.08 | -0.98 |
|  | (0.89) | (1.12) |
|  |  |  |
| Num. Words in Part 1 | 1.30*** | 1.45*** |
|  | (0.08) | (0.12) |
|  |  |  |
| Female | -0.33 | 1.88 |
|  | (0.98) | (1.14) |
|  |  |  |
| Age | -0.06 | -0.01 |
|  | (0.05) | (0.05) |
|  |  |  |
| Risk Loving | -0.05 | 0.25 |
|  | (0.16) | (0.20) |
|  |  |  |
| Constant | 22.19*** | 11.81*** |
|  | (2.93) | (3.59) |
| R^2^ | 124 | 147 |
| N | 0.713 | 0.538 |

*Note*: Dependent variable is number of words encoded in Part 2. The first model includes only pessimistic subjects who believe that the target is above the median belief of 30. The second model includes optimistic subjects who have below-median beliefs. For a few subjects, we have missing data on some control variables and so they drop out of the regressions. Significance levels: ^***^ = 1%; ^**^ = 5%; ^*^ = 10%.
